# Deciphering transcriptomic changes in chemobrain: a comprehensive review

**DOI:** 10.1186/s40478-025-02102-z

**Published:** 2025-08-30

**Authors:** Tusar Kanta Acharya, Yuan Pan, Peter M. Grace, Cobi J. Heijnen, Rajasekaran Mahalingam

**Affiliations:** 1 Laboratories of Neuroimmunology, Department of Symptom Research, University of Texas MD Anderson Cancer Center, Houston, TX 77030 USA; 2 Department of Neuro-Oncology, University of Texas MD Anderson Cancer Center, Houston, TX 77030 USA; 3 Cancer Neuroscience Program, University of Texas MD Anderson Cancer Center, Houston, TX 77030 USA; 4 Department of Psychological Sciences, Rice University, Houston, TX 77005 USA

**Keywords:** Chemotherapy, Chemo brain, CICI, Transcriptomics, Cognitive dysfunction

## Abstract

Chemotherapy-induced cognitive impairments (CICI), colloquially known as “chemobrain,” represents a profound and debilitating side effect experienced by a significant number of cancer survivors, impacting their memory, multitasking, and quality of life. This review critically evaluates the molecular mechanisms underlying CICI, with a particular focus on the insights gained from transcriptomic analyses. As cancer incidence rises globally, understanding the complex interplay between chemotherapy agents and their cognitive repercussions becomes increasingly vital. Key mechanisms implicated in CICI include blood-brain barrier disruption, neuroinflammation, and oxidative stress as a result of various chemotherapy treatments, such as doxorubicin, cisplatin, and paclitaxel. We delve into advanced transcriptomic methodologies including RNA sequencing, cDNA microarrays, and single-cell transcriptomics that elucidate the alteration in gene expression profiles associated with CICI and provide a deeper understanding of the underlying pathophysiological processes. Furthermore, we emphasize the importance of developing comprehensive single-cell atlases and employing spatial transcriptomics to uncover cellular heterogeneity and the spatial dynamics of gene expression across different brain regions. This review consolidates the existing literature on the transcriptomic profile of CICI, highlighting potential genes and pathways while suggesting future research avenues aimed at mitigating cognitive dysfunction. Ultimately, integrating transcriptomic findings with clinical insights is essential for the development of targeted, personalized interventions, thereby improving cognitive health and overall quality of life for cancer survivors dealing with long-term impacts of their treatment.

## Introduction

Cancer causes more than one in six deaths (16.8%), which poses a serious social, public health, and economic crisis in the twenty first century [1]. Cancer ranks as the second leading cause of mortality globally following cardiovascular diseases [2]. According to World Health Organization (WHO), cancer accounts for nearly 10 million deaths in 2020, or nearly one in six deaths. The incidence of cancer has shown an upward trend over time, largely attributed to population growth and aging [3, 4]. Despite this concerning rise, advancements in early detection, innovative therapies, and the adoption of multidisciplinary treatment approaches have contributed to a notable improvement in the survival rates of various cancer types. For instance, a combination of surgical interventions, chemotherapy, radiotherapy, and immunotherapy has significantly enhanced patient outcomes [5, 6, 7]. According to the American Cancer Society’s reports, the United States witnessed a surge in cancer survivors, reaching 18.1 million in 2022. Projections suggest this number will escalate to over 26 million by 2040, reflecting the increasing survival rates [8]. However, the widespread use of chemotherapy comes with a significant toll on patients as it often induces a myriad of adverse effects, ranging from acute to chronic [9, 10, 11, 12, 13, 14, 15, 16]. These side effects can profoundly impact patients’ quality of life, presenting challenges that extend beyond the cancer diagnosis itself.

Among these adverse effects, chemotherapy-induced cognitive impairment (CICI), often commonly referred to as “chemobrain,” has garnered significant attention [17, 18, 19]. CICI manifests as deficits in memory, attention, executive function, psychomotor function, visuospatial function and processing speed, affecting a considerable proportion of cancer patients [20, 21, 22, 23, 24, 25]. The prevalence of cognitive decline during and after chemotherapy is estimated to be as high as 70–75%, with approximately 50% of patients experiencing persistent cognitive impairments [26, 27, 28]. These enduring cognitive deficits have extensive ramifications, affecting various aspects of patients’ lives, including daily functioning, work performance, social interactions, and psychological well-being.

CICI is thought to arise from a complex interplay of mechanisms, including blood-brain barrier (BBB) disruption, neuroinflammation, and oxidative stress. Chemotherapy drugs such as docetaxel, cisplatin, carmustine, oxaliplatin, 5-fluorouracil, paclitaxel, doxorubicin, and methotrexate have been shown to influence BBB permeability, as well as promote neuroinflammation and oxidative stress. These changes can interfere with critical cellular pathways, potentially leading to brain cell damage [29, 30, 31, 32, 33, 34, 35, 36]. Moreover, chemotherapy has been associated with increased levels of proinflammatory cytokines such as interleukin 1 beta (IL-1β), interleukin 6 (IL-6), interleukin 8 (IL-8), and tumor necrosis factor alpha (TNF-α), as well as decreased antioxidant defense systems, exacerbating oxidative stress [37, 38, 39, 40, 41]. A recent study suggests that chemotherapy related cognitive impairment (CRCI) shares molecular similarities with age-related neurodegenerative disorders like Alzheimer’s and Parkinson’s disease. By comparing brain gene expression profiles from rodent models of CRCI with validated rodent models of Alzheimer’s and Parkinson’s disease, the study identified 165 overlapping genes, including 15 genes common to all three conditions. These shared genes highly similar to human and are mainly involved in changes in neural activity, neuroinflammation and cell cycle arrest [42]. Furthermore, impaired neurogenesis, myelin breakdown, and genetic predispositions are also shown to contribute to CICI [43, 44, 45, 46, 47, 48, 49], although the full mechanisms remain elusive. Overall, while these mechanisms shed light on the pathophysiology of chemobrain, further research is needed to fully understand the cellular mechanisms underlying this condition.

Considering these challenges, transcriptomics a comprehensive study of RNA transcripts within cells or tissues offers a promising avenue for unraveling the molecular underpinnings of CICI. Transcriptomic analyses, particularly in animal models, enable the profiling of gene expression patterns in response to chemotherapy, providing insights into the intricate molecular pathways implicated in cognitive dysfunction. By integrating findings from preclinical models and clinical studies, transcriptomic analyses hold the potential to elucidate the molecular signatures associated with CICI, identify novel therapeutic targets, and facilitate the development of more effective, tailored interventions for patients.

Despite the growing recognition of CICI’s clinical significance, there remains a gap in our understanding of its molecular mechanisms and the role of transcriptomics in elucidating these pathways. Therefore, this review aims to synthesize existing knowledge on the transcriptomic landscape of chemotherapy-induced cognitive impairment. By examining the current literature, we seek to shed light on the molecular alterations underlying CICI, explore the potential of transcriptomic analyses in identifying therapeutic targets, and outline future research directions aimed at mitigating the impact of cognitive dysfunction on cancer survivors’ long-term well-being.

## Transcriptome methods in chemotherapy-induced cognitive impairment

Understanding the molecular basis of CICI necessitates advanced techniques capable of deciphering intricate gene expression changes in neuronal tissue and cells. Transcriptomic methodologies have emerged as indispensable tools in this pursuit, offering a comprehensive view of the genetic landscape underlying CICI. In this section, we delve into the principles, applications, and nuances of transcriptome methods, including cDNA microarray-based transcriptomics, RNA sequencing (RNAseq), single-cell transcriptomics and spatial transcriptomics, within the context of CICI research.

**Fig. 1:**
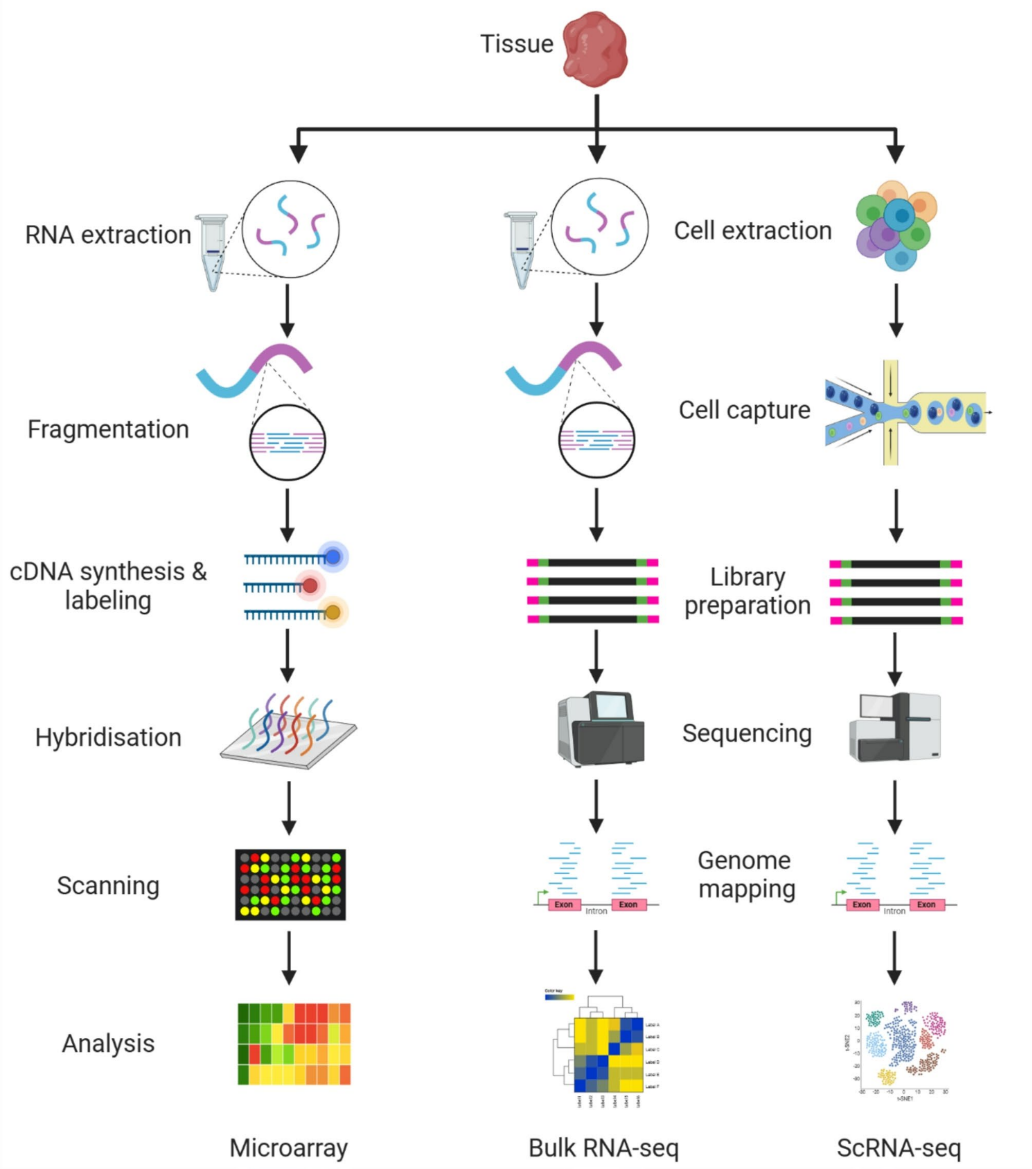
Schematic representation of workflow for three major transcriptomic techniques: microarray, bulk RNA-seq, and single-cell RNA-seq. (This figure has been prepared by the help of Bio render)

cDNA microarrays have been pivotal in profiling gene expression alterations associated with CICI [50]. This approach relies on the hybridization of fluorescent-labeled cDNA synthesized from RNA samples with probe oligonucleotides immobilized on microarray chips (Fig. 1) [51, 52, 53]. While cDNA microarrays offer high-throughput capabilities and cost-effectiveness, their reliance on known gene sequences poses limitations. Additionally, issues such as background noise and resolution constraints have been encountered. Nonetheless, cDNA microarrays continue to provide valuable insights into gene expression patterns relevant to CICI, particularly in elucidating the broader transcriptional landscape.

RNAseq represented a revolutionary shift in transcriptomic analysis, offering unparalleled sensitivity and resolution [54, 55, 56]. This technique involves converting RNA molecules into a cDNA library, followed by high-throughput sequencing to generate short reads (Fig. 1). These reads are subsequently mapped to a reference genome or assembled de novo, enabling the quantification and identification of transcribed genes. RNAseq’s ability to detect differential gene expression without prior knowledge of gene sequences has only begun to revolutionize CICI research. Moreover, RNAseq provides insights into post-transcriptional RNA processing events, including alternative splicing, which may play a role in CICI pathogenesis. The downstream analysis of RNAseq data, such as Gene Ontology (GO) enrichment and KEGG (Kyoto Encyclopedia of Genes and Genomes) pathway analysis, provides more valuable information about the biological processes and pathways involved in CICI, such as neuroinflammation, oxidative stress, and mitochondrial dysfunction, helping to uncover the molecular mechanisms underlying chemotherapy-induced cognitive impairment.

Single-cell transcriptomics has emerged as a transformative approach for dissecting the heterogeneity of cellular populations contributing to CICI [57]. By profiling gene expression at the single-cell level, this methodology offers unprecedented insights into the cellular diversity and dynamics underlying cognitive dysfunction. Single-cell transcriptomics typically involves isolating individual cell subsets, amplifying their RNA content, and subjecting it to high-throughput sequencing (Fig. 1). Although single-cell transcriptomics provides invaluable information about rare cell types and sub-populations, challenges such as detection thresholds for low-abundance RNAs and technical noise persist.

The wealth of data generated by transcriptomic analyses necessitates sophisticated computational tools for interpretation. Statistical algorithms and bioinformatic pipelines, tailored to each transcriptomic method, are employed to extract meaningful insights from complex datasets. Network analysis tools facilitate the visualization and exploration of gene interactions, shedding light on the molecular pathways implicated in CICI. Moreover, gene ontology and pathway enrichment analyses offer a systematic approach to deciphering the functional significance of differentially expressed genes in CICI.

## Transcriptomes in chemotherapy-induced cognitive impairment research

Since their introduction, transcriptomic studies of brain tissue and cultured brain cells isolated from mice, rats, or humans have provided fundamental insights into the gene expression profiles and transcriptional machinery of brain cells in CICI (Table 1). These studies often utilize multiple brain regions, including the hippocampus, cortex, choroid plexus, and whole brain, which encompass both neuronal and non-neuronal cell types. Large-scale single-cell transcriptomics analyses of human and mouse brains have also been developed [58, 59], leading to the creation of interactive online databases (atlas.brain-map.org) that widely characterize brain cell RNA expression. The transcriptional landscapes of human and mouse brains, accessible through these databases and transcriptomic analyses, allow for comparisons of gene expression across species and different tissues.

**Table 1:** Effects of various chemotherapeutics on cerebral areas and their correlation with neurological function

Drug	Drug quantity	Organism	Tissue/cells	Method	Comments	References
Adriamycin (ADR) or Doxorubicin	2 mg/kg	Mice	Hippocampus	mRNA expression by nano- string mouse immunology panel	Gene expression analysis of microglial and inflammatory markers from the mice hippocampus show upregulation of pro-inflammatory signatures in the ADR-treated brains	[60]
Bortezomib	1 mg/kg	Mice	Brain microvascular endothelial cells (bEnd.3)	RNA-seq	GO term analysis showed differential gene expression enriched in autophagy-lysosome pathway in bEnd.3 cells following BTZ exposure. RNA-seq analysis showed downregulated lysosome-associated genes and reduced *Tfeb* mRNA levels when bEnd.3 were exposed to BTZ	[61]
Cisplatin	2.3 mg/kg	Mice	Hippocampus	Bulk RNA-seq	Found that following cisplatin treatment, there were 24 down-regulated and 85 up-regulated genes.	[62]
Cisplatin	2.3 mg/kg	Mice	Pre-Frontal Cortex (PFC)	Bulk RNA-seq	RNAseq analysis identified the TR/RXR pathway as one of the top canonical pathways activated by administration of bexarotene to cisplatin-treated mice	[63]
Cisplatin	2.3 mg/kg	Mice	Hippocampus	Bulk RNA-seq	Transcriptome analysis by RNA-sequencing showed reversal of cisplatin-induced changes in the expression of about seven hundred genes in the hippocampus. Pathway analysis identified *Nrf2*-mediated response as the top canonical pathway.	[60]
Cisplatin	2.3 mg/kg	Mice	L3-L6 DRG	RNA-seq	Ingenuity pathway analysis indicates that genes that were downregulated by cisplatin and upregulated by IACS98287 largely mapped to neurological disease and organismal injury and abnormalities. Furthermore, many genes implicated in pain response were altered by cisplatin. Many transcripts encoding voltage-gated potassium channels were decreased by cisplatin	[64]
Cisplatin	2.3 mg/kg	Mice	Hippocampus	RNA-seq	In cisplatin-treated animals, functional enrichment analysis showed increased function related to neuronal degeneration, apoptosis, and necrosis over time. This indicated that cisplatin treatment is inducing neurotoxicity in the hippocampus	[65]
Cisplatin	2.3 mg/kg	Mice	Cortex	RNA-seq	Cisplatin treatment altered the expression of 1688 genes of which 868 genes were up-regulated, and 820 genes were down-regulated	[66]
Cisplatin	2.3 mg/kg	Mice	Meninges	RNA-seq	Cisplatin-treated mice triggered differential expression of 2433 genes (1619 upregulated/ 814 downregulated) in the meningeal transcriptome	[67]
Cyclophosphamide (CPP) and mitomycin C (MMC)	CPP: 400-1800 mg/m² MMC: 20 mg/m2	Mice	Hippocampus and Pre-Frontal Cortex	RNA-seq	Gene expression changes were most prominent in the PFC tissues of female mice 3 weeks after MMC treatment, and the gene expression response was much greater for MCC than CPP exposure. The PFC of females exposed to MMC at three weeks showed downregulated genes belonging to dopaminergic neurogenesis and oxidative phosphorylation pathway	[68]
Cyclophosphamide (CPP) and mitomycin C (MMC)	CPP: 200 mg/kg MMC: 5 mg/kg	Mice	PFC and hippocampal	RNA-seq	The transcriptomic analysis revealed no notable changes in gene expression in the PFC after exposure to CPP. In contrast, changes in gene expression in response to MMC treatment were evident three weeks after exposure, especially in female mice. Thirty-six genes were upregulated, and 166 genes were downregulated in female mice, while only 2 and 16 genes were upregulated and downregulated	[68]
Doxorubicin	10 mg/kg	Mice	Hippocampus	Bulk RNA-seq	Differentially expressed genes altered synaptic transmission and neurotransmitter function, neuronal health, and behavior related pathways	[69]
Doxorubicin	2 mg/kg,	Rat	Bone marrow Stem cells-exosomes	Next generation sequencing	Most abundant miRNA expressed in the exosomes of BMSCs showed that 17 of the 18 highly expressed miRNAs are most significantly associated with neurological and psychological disorders	[70]
Doxorubicin	5 mg/kg	Mice	Hippocampus	Single-nucleus RNA-seq analysis	Upregulation of genes following doxorubicin treatment were associated with neurodegeneration and cellular stress including *Apoe*,* Ttr*,* Cox6c*,* Col6a1*,* Crym*, and Ntng1 and downregulation of genes related to microglia homeostasis and synaptic organization including *Slco2b1*,* Fermt3*,* Prickle2*,* Dock3*,* Msn*,* Scamp5*,* Lrp1b*,* Tgfbr1*,* Rhoa*,* Fut8*,* Cadm2*, and *Pdlim5.*	[57]
FEC (5-fluorouracil, epirubicin and cyclophosphamide)	5-FU: 50 ug/ml epirubicin: 0.5 ug/ml	Human	Human T98G neuroglia cells	Transcriptome-wide RNA microarray	FEC deregulated 67 genes involved in decrease of development of neurons, 37 genes involved in development of the sensory system, 12 genes in extension of axons, and 3 genes in migration of neurons	[71]
Mithramycin (MTR)	0.1 mg/kg	male rats	Frontal cortex	Microarray chip gene expression analysis	MTR exposure was associated with the ribosome, lysosome, oxidative phosphorylation, notch signaling, glutathione metabolism, and TGF-beta signaling pathways	[50]
Methotrexate (MTX)	75 mg/kg	Mice	Choroid Plexus (ChP)	ScRNA-seq	Single-cell gene expression analysis revealed *Sod3* expression predominantly in epithelial and mesenchymal cells at the ChP in each ventricle in the brain	[72]
Paclitaxel	5 mg/kg	Mice	Whole brain	Spatial transcriptomics and ScRNA-seq	Differentially expressed genes showed increased senescence markers in capillary endothelial cells inducing neurovascular coupling dysfunction, blood-brain barrier disruption and neuroinflammation	[73]
Paclitaxel	5 mg/kg	Mice	Whole brain	ScRNA-seq	Single cell RNA-seq analysis revealed that senescent capillary endothelial cells exhibit downregulated pathways related to cell junction assembly and upregulated pathways associated with extracellular matrix remodeling and inflammatory signaling, including vitronectin and pleiotrophin.	[74]
Paclitaxel	4 mg/kg	Mice	Pre-Frontal Cortex (PFC)	RNA-seq	PTX induced 1755 differentially expressed genes in the PFCs of male and female mice. Among these 147 upregulated genes in female mice, 7 genes were upregulated by PTX, and 56 genes were downregulated by PTX. Meanwhile, 7 genes were upregulated by PTX, and 59 genes were downregulated by PTX amongst the 153 upregulated genes in male mice	[75]
Tamoxifen	2 mg/kg	Mice	Hippocampus	RNA-seq	Tamoxifen altered 669 genes in hippocampus. 647 genes were down-regulated, and 22 were up-regulated. Down regulated pathways were associated with cilium movement, microtubule assembly, regulation and transport motors (genes identified: *DNAI3*,* ODAD4*,* CFAP43*,* CFAP 206*,* ODAD2*,* FOXJ1*,* HYDIN*) while the upregulated pathways involved in negative regulation of dendritic spine morphogenesis (genes identified: *PREN*,* UBE3A*,* NGEF*,* EFNA1*,* NLGN3*,* DNM3*,* DTNBP1*,* NLGN1*).	[76]
Other chemotherapy Regimens		Human	Blood	RNA-Seq	GE analysis shows 93 patients with high AFI (Attentional Function Index) and 89 patients with low AFI (Attentional Function Index) out of 182 patients	[77]
Other chemotherapy Regimens		Human	CSF	Smart-seq2 RNA-sequencing protocol	Sequencing of CSF extracellular mRNA in all patients with fatigue revealed 76 genes differentially expressed compared to patients without fatigue. GO-term analysis suggested reduced apoptosis and autophagy as well as decreased production of cytokines involved in immune responses and immune cell migration	[78]

Transcriptomic analysis has provided unprecedented insight into the molecular fingerprint and gene expression profile of different brain cell types. These advancements underscore the potential of transcriptomic studies to elucidate the molecular underpinnings of CICI. Chemotherapeutic agents such as adriamycin, bortezomib, cisplatin, doxorubicin, tamoxifen and paclitaxel have been shown to induce transcriptomic changes that may contribute to cognitive dysfunction. By leveraging transcriptomic analyses, researchers can explore the gene expression alterations associated with these drugs, providing critical insights into the mechanisms driving chemobrain and identifying potential therapeutic targets. The following sections explain the transcriptomic changes induced by each drug and how these changes contribute to the development of chemobrain.

## Paclitaxel

Paclitaxel, a chemotherapeutic agent of the taxane class, is used to treat cancers such as breast, lung, and ovarian cancer [79]. Paclitaxel works by binding to microtubules and enhancing tubulin polymerization, resulting in cell-cycle arrest and apoptosis [80]. Despite its effectiveness in increasing progression-free and overall survival, paclitaxel treatment can cause peripheral neuropathy, emotional deficits, and cognitive impairments [31, 81, 82, 83, 84]. Paclitaxel may impact brain tissues directly, as demonstrated by its presence in the brain [31], and low concentrations can induce morphological changes in neurons [85, 86].

Ahire et al. [73] conducted a detailed single-cell RNA sequencing (scRNA-seq) study to understand the cellular and molecular changes in the brain following paclitaxel treatment. Their analysis revealed six distinct cell clusters in brain tissue, including endothelial cells, microglia, oligodendrocytes, neurons, astrocytes, and pericytes. Among these, endothelial cells showed the most significant changes. Paclitaxel treatment induced cellular senescence predominantly in capillary endothelial cells, a phenomenon that can compromise the blood-brain barrier (BBB) permeability and can contribute to neuroinflammation. Building on these findings, further spatial transcriptomics analysis showed that senescent cells were identified through the expression of the *Cdkn2a* gene which was most prevalent in the isocortex and white matter regions of paclitaxel-treated mice. This increased senescence corresponded with disrupted BBB integrity and elevated neuroinflammatory markers, indicating a critical role of endothelial cell senescence in paclitaxel-induced cognitive impairment. To counter these effects, the study explored the use of senolytic drugs, specifically navitoclax (ABT263), which are designed to selectively eliminate senescent cells. Treatment with navitoclax in paclitaxel-treated mice led to a significant reduction in senescent cell burden. This intervention restored BBB integrity, reduced neuroinflammation, and improved cognitive performance, as evidenced by behavioral tests. These findings highlight the potential of senolytic therapy as a possible strategy to protect against paclitaxel-induced neurovascular damage and cognitive deficits. Further supporting these findings, Patai et al. demonstrated that paclitaxel induces senescence of capillary endothelial cells, which disrupt BBB integrity and promotes neuroinflammation. Single cell RNA sequencing analysis of paclitaxel treated mouse brains confirmed the presence of endothelial senescence cells characterized by downregulated pathways involved in cell junction assembly and upregulated pathways related to extracellular matrix remodeling, inflammatory signaling, including Vitronectin (VTN) and Pleiotrophin (PTN) pathways. Further cell-cell communication analysis revealed reduced junctional adhesion molecule (JAM) signaling, highlighting endothelial senescence as a key driver of BBB dysfunction in paclitaxel associated cognitive impairments [74].

Complementing this research, Liang et al. [75] focused on the transcriptional changes in the prefrontal cortex (PFC) of mice following paclitaxel treatment. Using RNA sequencing, they identified 1,755 differentially expressed genes (DEGs) in the PFC of paclitaxel-treated mice compared to controls. These DEGs were analyzed to understand their roles and the pathways they influence, Gene Ontology (GO) enrichment analysis showed that the DEGs were involved in various biological processes. KEGG pathway analysis further revealed that these DEGs were significantly associated with synaptic transmission and neuroplasticity. Notably, paclitaxel treatment led to changes in the expression of genes involved in synaptic transmission. There was an increase in the expression of genes related to inhibitory synaptic transmission, such as *Gabrb1* and *Gabrg1*, which encode subunits of the *GABA*_*A*_ receptor. This suggests a shift towards enhanced inhibitory signaling in the PFC, which could contribute to the cognitive and emotional impairments observed in paclitaxel-treated individuals. Additionally, the expression of serotonin receptor genes (*5-HTR1A* and *5-HTR2C*) was altered by paclitaxel treatment. These receptors play critical roles in regulating mood and cognitive functions, and their dysregulation is associated with depression, anxiety, and cognitive deficits.

Overall, these studies provide valuable insights into the cellular and molecular mechanisms underlying paclitaxel-induced cognitive impairment. The findings indicate that paclitaxel-induced changes in serotonin signaling may underlie some of the mood and cognitive symptoms seen in patients. By identifying specific gene expression changes and pathways affected by paclitaxel, these findings pave the way for targeted therapeutic strategies to mitigate the adverse cognitive effects of chemotherapy. The integration of these findings highlights the complex transcriptional landscape changes induced by paclitaxel but underscores the potential of targeted therapies, such as senolytics and interventions aimed at restoring synaptic balance, to improve cognitive outcomes for cancer patients undergoing chemotherapy.

## Bortezomib

Bortezomib is a first-in-class proteasome inhibitor widely used to treat mantle cell lymphoma and multiple myeloma [87]. It works by blocking the activity of the 26 S proteasome, thereby inhibiting the ubiquitin proteasome pathway, leading to the accumulation of misfolded proteins within the cell [87]. Although bortezomib poorly crosses the blood-brain barrier (BBB), clinical and imaging studies have shown that it can induce a range of neurological symptoms such as epilepsy, confusion, loss of consciousness, paralysis, and numbness [61, 88, 89, 90].

Lu et al. employed bulk RNA-seq to investigate the effects of bortezomib on brain microvascular endothelial cells (bEnd.3 cells) [61]. Their analysis revealed significant upregulation and downregulation of numerous genes in response to bortezomib treatment. Gene Ontology (GO) term analysis highlighted that the differential gene expression was notably enriched in the autophagy-lysosome pathway. Specifically, genes involved in lysosome biogenesis, such as *Tfeb*, *Lamp1*, and *Lamp2a*, were downregulated. Bortezomib exposure impaired autophagic flux, as evidenced by the increased expression of LC3-II and SQSTM1/p62 proteins, indicating the accumulation of autophagic cargo due to inhibited autophagic clearance. Additionally, the RNA-seq data showed a significant decrease in *Tfeb* mRNA levels and reduced nuclear translocation of TFEB protein in bortezomib-treated cells, impairing lysosome biogenesis and proteostasis maintenance [61].

The study further revealed that bortezomib treatment elevated the expression of the inflammatory cytokine *Il23a* at both the mRNA and protein levels in human brain microvascular endothelial cells (HBMECs) and in the serum of patients. This upregulation of *Il23a* triggered microglial activation, leading to increased phagocytosis of neuronal synaptic proteins. The IL23A-STAT3 signaling pathway was identified as a key mediator in this process, with bortezomib inducing STAT3 phosphorylation and nuclear translocation, thereby upregulating *Il23a* transcription. These findings highlight a critical role for the TFEB-STAT3-IL23A pathway in bortezomib-induced neuroinflammation and cognitive impairment. Furthermore, the study demonstrated that digoxin, a pharmacological agent, could reverse these effects by enhancing TFEB nuclear translocation and autophagic flux, ultimately ameliorating bortezomib-induced cognitive dysfunction. Overall, this transcriptomic analysis provides a comprehensive understanding of the molecular mechanisms by which bortezomib induces CICI, emphasizing the disruption of autophagy-lysosome pathways and the activation of proinflammatory signaling [61].

This phenomenon suggests that bortezomib may disrupt protein homeostasis in the brain, potentially through mechanisms involving autophagy inhibition and inflammatory responses, leading to neurotoxicity and cognitive dysfunction.

## Mithramycin

Mithramycin (MTR) is an antineoplastic antibiotic that interferes with gene transcription by competing with transcription factors of the specific protein (Sp) family, such as *Sp1*, on GC-rich DNA binding sequences [91]. Sp1 is crucial for the expression of many genes, including those involved in early development and cognition, like reelin, *Mao* (A and B), *NMDA* receptor subunits, *GABA*_*A*_ receptor, and oxidative phosphorylation system proteins [50]. By disrupting these pathways, mithramycin can induce changes in gene expression that affect brain development, potentially leading to long-term CICI.

In their study, Asor et al. used microarray to investigate the transcriptomic effects of early life mithramycin treatment in a rat model. Initially, mithramycin treatment altered the expression of only a limited number of genes (90 genes), but three months later, over 1,000 genes were differentially expressed. Gene Ontology (GO) analysis revealed that long-term changes involved cellular organization, development, and metabolic processes. Pathway analysis using the Database for Annotation, Visualization, and Integrated Discovery identified significant disruptions in the ribosome, lysosome, oxidative phosphorylation, Notch signaling, glutathione metabolism, and TGF-beta signaling pathways. Specific genes, such as *Ndufv2*, *Ywhae*, and *Dab1*, showed significant downregulation and were linked to the observed cognitive deficits and anxiety-like behavior in adult rats [50].

Key findings included a negative correlation between the expression of *Ndufv2*, which encodes a subunit of mitochondrial complex I, and anxiety levels, as well as working memory performance. *Ywhae*, encoding the 14-3-3 ε protein involved in signaling pathways and associated with neuropsychiatric disorders, also showed significant downregulation. *Dab1*, a crucial gene for neuronal positioning and Reelin signaling, was similarly affected. The study’s transcriptomic analysis demonstrated that mithramycin treatment during critical developmental periods induces long-lasting changes in gene expression, leading to cognitive and behavioral impairments. These findings underscore the importance of understanding chemotherapy’s long-term effects on brain function and highlight potential targets for mitigating CICI [50].

## Methotrexate

Methotrexate (MTX) is a chemotherapeutic and folate analog widely used in treating brain cancers and other type of cancers. Methotrexate has the ability to penetrate the central nervous system (CNS) [92]. Its primary mechanism of action involves the inhibition of nucleic acid synthesis, leading to the death of dividing tumor cells [92]. However, Methotrexate treatment is associated with significant neurological side effects, up to 75% of cancer patients treated with Methotrexate experience symptoms such as compromised fine motor skills, attention deficits, acute memory impairment, and permanent cognitive dysfunction [72, 93]. These side effects have become increasingly relevant as advancements in cancer treatment have reduced mortality rates, shifting focus towards the quality of life for cancer survivors. Gibson et al., 2019 demonstrated that administration of Methotrexate promotes microglia to become persistently activated which in turn induces activation of astrocytes dependent of inflammatory microglia. Microglia depletion restores oligodendroglial lineage dynamics, myelin microstructure and cognititve behavior following Methotrexate treatment. These findings directs the association of Methotrexate in tri-glial dysregulation and depletion of inflammatory microglia can be used as a target for CICI [94].

The study by Jang et al. utilized transcriptomic analysis to investigate the molecular pathways and genes affected by Methotrexate treatment in the brain, contributing to chemobrain [72]. Their key findings highlighted broad metabolic damage to the choroid plexus (ChP)-cerebrospinal fluid (CSF) system, including decreased secretion of the antioxidant enzyme extracellular superoxide dismutase 3 (*Sod3*) into the CSF. This reduction in *Sod3* was associated with increased oxidative stress and cognitive impairment. By employing adeno-associated viral (AAV) gene therapy to overexpress *Sod3* in the ChP, the researchers were able to protect the brain from oxidative damage and prevent cognitive deficits in Methotrexate-treated mice. The transcriptomic analysis revealed significant alterations in oxidative metabolism, particularly in the glutathione (GSH/GSSG) redox balance, indicating that oxidative stress plays a pivotal role in Methotrexate-induced neurotoxicity. These findings suggest that targeting the ChP-CSF axis with antioxidant therapies could be a promising strategy to alleviate chemobrain and improve the quality of life for cancer patients undergoing Methotrexate treatment [72].

The underlying mechanisms of Methotrexate-induced CNS toxicity is complex, involving persistent dysregulation of various brain cells and oxidative stress, which suggests potential therapeutic targets to mitigate CICI.

## Doxorubicin

Doxorubicin is a chemotherapeutic agent widely used in several types of cancer [95]. The widespread use of the drug is associated with many cytotoxic effects. The most impacted organ is the brain. Treatment of doxorubicin as a prime chemotherapy causes brain dysfunction, cognitive impairment, memory loss which impose severe distress among the patients [96]. The precise mechanisms by which doxorubicin induces cognitive impairment remain incompletely elucidated. Recent discoveries regarding the transcriptomics data of various brain regions in animals treated with doxorubicin provide insights into the underlying mechanism involved in it.

The mitochondrion is a crucial organelle which is impacted by chemotherapy-induced toxicity [97]. Mitochondrial health is one of the important parameters of the adverse effects of doxorubicin. Cavalier et al. 2021 conducted a transcriptomic analysis of the brain hippocampus of mice treated with doxorubicin and Mito Q as a mitochondrial therapeutic [69]. The key findings of this group were that Mito Q prevented the hippocampus transcriptomic alterations induced by doxorubicin. The transcriptomics data postulated that animals treated with only doxorubicin have increment of ~ 30 transcripts and reduction of ~ 70 transcripts. Whereas Mito Q treatment partially rescued the transcriptomics changes; 9 significantly increased transcripts and 22 significantly decreased transcripts. The genes affected by doxorubicin but sustained by Mito Q were associated with the health and function of neurons and mitochondria. The GO (Gene Ontology) analysis indicated that doxorubicin treatment altered the neuronal function/health and behavior, and Mito Q treatment partially inhibited these transcriptional networks. More precisely the treatment of doxorubicin decreased neurotransmitter transport, synaptic transmission, neuron differentiation, regulation of neurotransmitter levels, synaptic vesicle exocytosis and increased the regulation of axonogenesis, regulation of dendrite morphogenesis, post-synaptic neurotransmitter receptor activity. However, doxorubicin + Mito Q treatment showed less significant alteration in neuronal function and behavior related transcriptomics, increased genes response to glucose, neuron axonogenesis, cell communication and cell signaling [69].

To alleviate the cognitive dysfunction induced by doxorubicin-induced chemotherapy, McAlpin et al. administered a histone deacetylase 6 (HDAC6) enzyme inhibitor to animals treated with doxorubicin [57]. HDAC6 is a zinc dependent cytoplasmic enzyme consisting of two functional catalytic domains. The major function of HDAC6 is to deacetylate α-tubulin and HSP90α and dysregulation of the activity of this enzyme leads to a myriad of diseases such as cancer, neurodegenerative disease and autoimmune response [98]. Notably, recent reports also support that inhibiting HDAC6 by a blood-brain barrier permeable drug ACY-1083 reversed the cisplatin induced CICI by reversing the bioenergetics of synaptosomal mitochondria [99]. McAlpin et al. demonstrated the effect of the HDAC6 inhibitor in doxorubicin induced CICI. The authors detected the alteration in the transcriptome profile by performing single nucleus RNA-seq of the hippocampal tissue six weeks after the treatment with ACY-1083 and doxorubicin. The authors have identified differential regulation of 135 common genes expressed by microglia in the set of treatments with both doxorubicin and doxorubicin + ACY-1083. Out of 135 common genes 113 genes altered by doxorubicin treatment were reversed with ACY-1083 treatment. The genes upregulated during the treatment of doxorubicin were associated with neurodegeneration, and cellular stress such as *Apoe*, *Ttr*, *Cox6c*, *Col6a1*, *Crym*, and *Ntng1*, and all these genes were downregulated by the treatment with ACY-1083. On the contrary, genes linked to microglia homeostasis and synaptic organization, such as *Slco2b1*, *Fermt3*, *Prickle2*, *Dock3*, *Msn*, *Scamp5*, *Lrp1b*, *Tgfbr1*, *Rhoa*, *Fut8*, *Cadm2*, and *Pdlim5*, were downregulated by doxorubicin treatment and upregulated by ACY-1083 treatment. Most important observation was ACY-1083 increased canonical microglia homeostasis genes such as *Cx3cr1*, *Tmem119*, *Fcrls*, *Hexb*, *Tgfbr2*, *P2ry12*, *Foxo3*, *Sall1*, and *Gpr34*, which were not altered by doxorubicin. To further comprehend the study’s findings, HDAC6 inhibition in the hippocampus areas of doxorubicin-treated animals in a long-term CICI paradigm promotes homeostatic microglia gene expression while also reversing the altered genetic signature in microglia [57].

Breast cancer is one of the prevalent and aggressive cancers people suffer in united states. Advancements in diagnosis and treatment techniques have increased survivability despite high incidence rates. However, cancer survivors experience an array of chemotherapy-induced side effects that impact their lifestyle [100]. Among several types of chemotherapy regimens, the most widely used recent breast cancer regimen is a combination of doxorubicin, cyclophosphamide, and docetaxel (the TAC regimen). Though this therapy increases the survivability of the patients but induce cognitive impairments. TAC treatment decreases the spatial memory retention as it decreases the dendritic complexity of arborization in the dentate gyrus region of the hippocampus. TAC also downregulates the key metabolic and signaling pathways associated with cognitive impairment [101]. Therefore, substances that have anti-inflammatory, anti-cancer, and antioxidant properties make effective combinations with chemotherapy to treat CICI. Among several antioxidant compounds, PL is an alkaloid compound of long pepper *Piper longum* L. has been extensively explored as an anti-cancer, antidepressant and anxiolytic [102]. In recent report PL was used as a co-therapeutic agent with TAC to evaluate the hippocampal-dependent social memory in C57BL/6J female mice [103]. During the treatment regimen with TAC and PL the changes in mRNA expression were evaluated. Comparing the TAC, PL, and TAC/PL treatments to the control, a reduction in *Nrf2* (nuclear factor erythroid 2-related factor 2) was observed. That suggests this regimen’s *Nrf2* activation also impacted the antioxidant response element (ARE) mRNA levels. The expression of the glutamate cysteine ligase enzyme catalytic subunit (GCLC), NADPH quinone dehydrogenase 1 (*Nqo1*), Heme oxygenase 1 (*Hmox1*), thioredoxin reductase 1 (*Txnrd1*) were differentially altered. The addition of PL in the regimen boosts the expression of these antioxidant enzymes in the TAC-treated condition, however in some cases it is less than the DMSO [103]. TAC treatment regimen also alters the mRNA expression of NMDA subunits (*Grin1*, *Grin2a*, *Grin2b*) and AMPA subunits (*Gria1*, *Gria2*). The *Grin1* mRNA expression remained unchanged, while *Grin2a* and *Grin2b* expression was reduced by PL and PL/TAC treatment. Contrarily, *Gria2* mRNA levels were elevated by the PL and TAC therapy, whereas Gria1 expression remained unchanged [103].

In another way to address the doxorubicin mediated chemobrain, El-Derany et al. used bone marrow derived mesenchymal stem cells (BMSCs) and their derived exosomes (BMSCs-Exo) in doxorubicin-induced chemobrain in rat models. They inferred that the BMSCs and BMSCs-Exo restored the doxorubicin-induced cognitive dysfunction by reducing hippocampal neurodegeneration and neural demyelination [70]. They performed RT-qPCR analysis of total RNA isolated from hippocampus. The results suggested that the expression of neural myelination genes mainly Opalin (Oligodendrocytic paranodal loop protein), Olig2 (Oligodendrocyte differentiation marker) were significantly decreased in doxorubicin treated chemobrain condition whereas the expression levels of these genes significantly increased in the BMSC- and BMSCs-Exo-treated groups. This indicates that the BMSC and BMSCs-Exo treatment reduce demyelination and induce remyelination. Furthermore, BMSC and BMSCs-Exo treatment also restored the expression level of hippocampal FGF-2 (fibroblast growth factor-2) and upregulated the hippocampal synaptic growth factors depleted in doxorubicin-induced chemobrain. Moreover, the expression of genes involved in Wnt and hedgehog signaling pathway were also checked. It showed that *Wnt3a*, *Wnt7b*, and *Fzd1* genes involved in WNT pathway rescued in the BMSC and BMSCs treatment which were reduced during doxorubicin induced chemobrain. Similarly, the reduction of the genes involved in Hedgehog signaling such as *Shh*, Ptch1 receptor, transcription factor *Gli1* in doxorubicin treated condition reversed upon BMSC or BMSCs-Exo treatment [70]. This study assessed the potential protective effects of BMSCs (bone marrow-derived mesenchymal stem cells) and BMSCs-Exo (extracellular vesicles derived from BMSCs) against chemobrain caused by doxorubicin, a routinely used cytotoxic drug.

Cognitive impairments are linked to increased microglial inflammation in the brain, and targeting microglia may help alleviate chemobrain. Using a rodent model of chronic adriamycin (ADR) also called doxorubicin hydrochloride treatment, Allen et al. 2019, tested two strategies (1) microglia depletion with the CSF1R (colony stimulating factor-1 receptor) inhibitor PLX5622 and (2) treatment with iMG (human induced pluripotent stem cell-derived microglia)-derived extracellular vesicles (EVs) and checked the alteration in cognitive impairment [104]. In the first strategy, PLX5622 effectively prevented adriamycin-induced cognitive deficits by depleting activated microglia. In the second, iMG-EVs restored cognitive function and reduced microglial activation in adriamycin-treated mice [104]. Analyses of gene expression revealed enhanced pro-inflammatory markers in the adriamycin-treated brain including *IL-6*,* IL-4*,* IL11ra1*, *Tnfsf13b* and *Cfi*. Furthermore, PLX5622 treatment reduced inflammatory gene expression levels in ADR-treated brains. Overall, blocking CSF1R led to less neuroinflammation in the brains of animals treated with ADR, which was linked to better cognitive function [104].

## Cisplatin

Cisplatin is a commonly used chemotherapy medication. It has been employed in the therapy of various types of cancer, including bladder, head and neck, lung, ovarian, and testicular tumors [105]. Cisplatin is considered one of the most effective chemotherapy medications used in cancer treatment. In 1978, the first platinum compound for cancer treatment was approved by the U.S. Food and Drug Administration (FDA) [106]. The mechanism of action of cisplatin has been associated with its capacity to form crosslinks with purine bases on DNA, which disrupt DNA repair pathways, inflict damage to DNA, and ultimately trigger apoptosis in cancerous cells [105]. Cisplatin treatment causes cognitive dysfunction and sensory and motor abnormalities associated with reduced myelin density and complexity in the cingulate and sensorimotor cortex [63]. To reduce the severity of the treatment regimen several investigators have identified certain molecules which can improve the chemobrain impact mediated by cisplatin. In search of a way to reverse these cisplatin-induced ailments, Chiang et al. 2020, examined the effect of the RXR (Retinoid X receptor) agonist bexarotene [63]. The main aim of their study was to identify whether bexarotene reverses the white matter alterations induced by cisplatin treatment. The data demonstrated that administration of only 5 daily doses of bexarotene after completion of cisplatin treatment is sufficient to normalize cognitive function, sensorimotor performance, and myelin (ultra)structure. RNAseq analysis of the prefrontal cortex (PFC) of the mice treated with cisplatin followed by bexarotene gives insights into the pathways activated by bexarotene treatment. Bexarotene treatment altered differential expression of 713 genes (172 down and 541 up). Bexarotene reversed the effect of cisplatin of five of these genes, *Lcn2*, *Hspa1a*, *Dusp5*, *Tbcid4*, and *Cxcl12*. Pathway enrichment analysis of the upregulated genes showed the activation of the neuregulin Signaling pathway, which is implicated in myelination and synaptic function. Similarly, the netrin pathway that is involved in providing axonal guidance cues and myelin maintenance was also predicted to be activated in mice treated with cisplatin and bexarotene. Other pathways affected were G-protein coupled receptor signaling, and axonal guidance Signaling. Regulator analysis showed five out of top 10 upstream regulators driving transcriptional changes in cisplatin + bexarotene treated mice were related to the RXR network, namely, *Rxra*, *Thra*, *Pgr*, *Ppara*, *Pparg*. Functional enrichment analysis of the up-regulated genes showed the development of neurons, neuritogenesis, and myelination of the nervous system [63].

Administering mitochondria derived from human mesenchymal stem cells to mice treated with cisplatin through the nasal route improved cognitive function by repairing the structure of myelin in the cingulate cortex and reversing synaptic loss in the hippocampus [60]. The underlying synaptosomal mitochondrial defects were also reversed by nasal mitochondrial administration. Transcriptomic analysis by RNA-sequencing revealed administration of mitochondria to cisplatin-treated mice reversed the cisplatin-induced change in expression of 676 genes. Top anti-correlated genes include *Nfe2l1*, *Atp6qp1* and *Apoa2*. Pathway prediction indicates that the activation of the *Nrf2*-mediated response may control antioxidant proteins to reduce oxidative damage. Rictor, a regulatory protein and sub-component of the mammalian target of rapamycin complex 2 (*mTORC2*), was projected to be activated as an upstream regulator in mice treated with cisplatin and administered mitochondria. Comparative pathway analysis comparing cisplatin-treated and PBS mice showed that mitochondria-induced transcriptome modulation was unique to cisplatin therapy and not a general impact on healthy mice [60].

Oliveros et al. 2022, discovered significant increase in the adenosine A2A receptor (*A2ar*) and its downstream effectors, cAMP and *Creb*, in the adult mouse hippocampus [62]. FDA-approved *A2ar* antagonist KW-6002 effectively prevented the detrimental impacts of cisplatin on the proliferation of neural progenitor cells and dendrite morphogenesis of adult-born neurons. It also improved memory and reduced anxiety-like behavior, without any impact on tumor growth or the anti-tumor effects of cisplatin [62]. After cisplatin treatment, RNA sequencing from the adult mouse hippocampus revealed 24 down-regulated and 85 up-regulated genes. Among the top five genes elevated by cisplatin without influencing other adenosine receptor subtypes was the *A2ar* gene. Increased *A2ar* correlated to biological pathways linked with long-term potentiation, learning, memory, and cognition, as well as neural development, morphogenesis, and proliferation, suggesting that *A2ar* dysregulation by cisplatin may underlie cisplatin-induced cognitive impairment, according to Ingenuity Pathway Analysis. This study inferred that cognitive impairment caused by cisplatin is mostly due to dysregulation of *A2ar* signaling. Specifically, cisplatin-induced neurogenesis defects are considerably mitigated by KW-6002-mediated *A2ar* antagonism [62].

Ma et al. [64] did a similar approach that specifically examined dual leucine zipper kinase (DLK), a crucial protein that plays a role in axonal degeneration and facilitates the neuronal response to damage. They used a novel brain-penetrant antagonist of DLK, IACS98287 in the background of cisplatin treatment. Concurrent administration of IACS98287 with cisplatin inhibits mechanical allodynia, loss of intraepidermal nerve fibers in the hind paws, cognitive impairments, and disruptions in brain connectivity in mice, despite not impacting the anticancer effects of cisplatin [64]. The RNA sequencing analysis of the DRGs from mice treated with IACS98287 and cisplatin revealed significant changes in the expression of 1689 genes. The results indicate an elevated level in DLK signaling activity in the DRGs during cisplatin treatment. Out of the genes that showed differential expression, a total of 582 genes were significantly changed by both cisplatin alone and by the combination of IACS98287 and cisplatin. Among these 582 altered genes, 504 genes showed downregulation in response to cisplatin treatment and upregulation when co-administered with IACS98287. Conversely, 72 genes showed upregulation upon cisplatin treatment but downregulation when IACS98287 was co-administered. The ingenuity pathway reveals that genes that were upregulated by IACS98287 and downregulated by cisplatin are mainly associated with neurological disorders and organismal damage and abnormalities. On the contrary, genes that were upregulated by cisplatin and downregulated by IACS 98,287 were mostly associated with inflammatory response pathways. Cisplatin reduced the expression of several voltage-gated potassium channel transcripts (*Kcnb1*, *Kcnc2*, *Kcnc3*, *Kcnc4*, *Kcnh2*, *Kcnk3*, *Kcnk12*, *Kcnn2*, *Kcnq2*, and *Kcns1*), while IACS98287 attenuated these changes. The expression of the Nogo protein (*Rtn4*) and its receptor (*Rtn4r*), which are associated with neuropathic and inflammatory pain disorders, was decreased by cisplatin and increased by IACS98287. The administration of cisplatin resulted in the upregulation of several leukocyte markers including *Itgam*, *Treml2*, *Il18rap*, *Clec5a*, *Cd33*, *Sell* (selectin L), *Il17rb*, and the cytokine *Il34*. Whereas IACS98287 downregulate these genes. Collectively, the data indicate that inhibiting DLK has a widespread influence on the transcriptome changes caused by cisplatin in DRG [64].

Another crucial study aimed at mitigating the cognitive impairment caused by cisplatin chemotherapy. In their study, Chiu et al. (2018) administered mesenchymal stem cells (MSCs) through the nasal cavity to mice that had cognitive abnormalities caused by cisplatin [107]. The administration of nasal MSC therapy completely resolved the cognitive impairments in both males and females. MSCs also restored the cortical myelin damaged by cisplatin. RNA sequencing revealed changes in the hippocampal transcription patterns seven days after the last cisplatin treatment. These changes resulted in altered expression of 390 genes when compared to the control group that received saline. Furthermore, there were 1335 genes that exhibited differential expressions when comparing the group treated with cisplatin and MSCs to the group treated with cisplatin alone. 105 genes that showed differential expression in response to cisplatin treatment also exhibited changes in expression when MSCs were administered to cisplatin-treated animals. Among the 105 genes that overlap, 45 showed an increase in expression due to cisplatin but a decrease in reaction to MSC. Conversely, 30 genes exhibited a drop in expression due to cisplatin but an increase in response to MSC. The pathway analysis showed that pathways such as “mitochondrial dysfunction” and “oxidative phosphorylation” altered in the expression of genes involved in bioenergetic pathways. MSC administration resulted in alterations in the expression of 29 nuclear encoded genes associated with mitochondrial function in mice treated with cisplatin (27 upregulated and 2 downregulated). The expression of mitochondrial genes that encode complex I, complex III, and complex IV was identified as altered by cisplatin and MSC treatment, while the expression of mitochondrial genes that encode ATP synthesis remained unaltered. Also, the treatment with MSCs increased the expression of one of the genes that encode mitochondrial ribosomal RNA [107].

Like the previous report, treatment of MSC-derived small extracellular vesicles (sEVs) could restore cisplatin-induced cognitive impairments and brain damage investigated by Milutinovic et al., 2023 [65]. Results showed abnormalities in mitochondrial morphology, loss of white matter, and synaptic integrity in the hippocampus due to cisplatin treatment were restored by sEVs treatment. The RNA sequencing analysis of the hippocampus revealed that there were 1804 genes that showed differential expression 24 h after the first dosage of sEVs, and a total of 1573 genes showed differential expression 24 h after both doses of sEVs. A functional enrichment study treated with cisplatin revealed a progressive rise in the functions associated with neuronal degeneration, apoptosis, and necrosis. These findings suggest that the administration of cisplatin is causing damage to the hippocampus. Following the delivery of two sEVs, there was a considerable enrichment in functions linked to neuronal growth, differentiation, and proliferation. The expression of genes associated with neuronal degeneration and damage was reduced. Treatment with small extracellular vesicles (sEV) in animals treated with cisplatin activates genes involved in the repair and development of neurons in the hippocampus area.

To treat and prevent neurotoxicities resulting from cisplatin, the chemical is utilized clinically. MRS5980 is an agonist of the A3 adenosine receptor (AR) subtype. MRS5980 effectively mitigated the cognitive damage caused by cisplatin, including reduced executive function and poor spatial and working memory. It also alleviated sensorimotor impairments and neuropathic pain, including mechanical allodynia and spontaneous pain, in both males and females [66]. An RNA-seq analysis was conducted on cortex samples collected 4 h after the final dosage of cisplatin, with or without MRS5980. The results showed that cisplatin therapy caused changes in the expression of 1688 genes. Out of these genes, 868 were up-regulated and 820 were down-regulated by cisplatin. The co-administration of MRS5980 with cisplatin resulted in altered expression of 528 genes, relative to cisplatin alone. Among these genes, 246 were up-regulated and 282 were down-regulated. Conducting a biological process enrichment analysis on the 246 genes that showed increased activity in the group treated with cisplatin + MRS5980, compared to cisplatin alone, revealed pathways primarily related to the regulation of translation and development (specifically Wnt signaling and stem cell differentiation), as well as phospholipid biosynthesis. Cisplatin affected the expression of 34 mitochondrial-related genes. Co-administration of MRS5980 blocked most of these alterations. MRS5980 completely averted cognitive impairments and structural harm to the brain caused by cisplatin. However, MRS5980 only prevented the alteration of 164 genes out of the 1688 genes that were changed by cisplatin alone. Functional enrichment analysis revealed that neuronal cell death, apoptosis, and necrosis were the pathways with the most prominent increase after cisplatin treatment without MRS5980. Genes associated with cellular stress, senescence, and hypoxia are reduced by injection of cisplatin + MRS5980. On the other hand, NOTCH1 signaling, and chromatin modification/organization-related genes are elevated by administering MRS5980 in conjunction with cisplatin. This research provides more evidence that MRS5980’s positive effects are partially mediated by its ability to activate repair pathways [66, 67].

The neurotoxicities induced by cisplatin can be efficiently reversed by nasally administering mesenchymal stem cell-derived mitochondria that have been coated with dextran-triphenylphosphonium polymer [67]. Coated mitochondria were shown to alter the expression of over 2400 genes that control immunological, neuronal, endocrine, and vascular pathways in the meninges of mice treated with cisplatin. Administering coated mitochondria nasally effectively cured the cognitive abnormalities caused by cisplatin. The reversal of these neuropathologies was linked to the resolution of impairments in myelination, synaptosomal mitochondrial integrity, and neurogenesis caused by cisplatin.

## Cyclophosphamide and mitomycin C

Kovalchuk et al. 2016, investigated the impact of two cytotoxic chemotherapy medications, cyclophosphamide (CPP) and mitomycin C (MMC), on the transcriptome and epigenetic alterations in the PFC and hippocampus regions of mice. The administration of CPP and MMC therapies resulted in significant and specific changes in gene expression profiles, both in terms of sex and brain area. The most significant alterations in gene expression were observed in the PFC tissues of female mice three weeks following treatment with MMC. Furthermore, the gene expression response was significantly more pronounced for MMC exposure compared to CPP exposure. The transcriptome study showed that there were no significant alterations in gene expression in the PFC following exposure to CPP. However, three weeks following exposure, particularly in female mice, alterations in gene expression were noticeable in reaction to MMC treatment. In female mice, the expression of 36 genes increased and the expression of 166 genes decreased after exposure to MMC. In contrast, in male mice, only 2 genes increased in expression and 16 genes decreased in expression after MMC exposure. Unlike the PFC, the hippocampal tissue of female mice showed essentially no differently expressed genes three weeks after MMC: only one gene, (predicted gene *EG545*), was elevated in male mice. Therefore, the alterations in gene expression caused by MMC were exclusive to certain regions of the brain, specifically the PFC and not the hippocampus [68].

The Gene Ontology (GO) analysis detected genes involved in the Notch signaling pathway and in neural crest cell development downregulated in the PFC of female mice subjected to MMC and the olfactory receptor activity upregulated. Genes related to the oxidative phosphorylation pathway and dopaminergic neurogenesis were found to be downregulated in the PFC of females that were administered MMC [68].

## FEC (5-fluorouracil, epirubicin and cyclophosphamide)

Human clinical investigations have shown that adaptogens or plant extracts have neuroprotective effects and can improve cognitive functions. Seo et al. 2019, evaluated the impact of specific adaptogenic herbal extracts on the alterations in transcriptome-wide RNA microarray profiles of neuroglia cells induced by chemotherapy regimen FEC (fixed combination of 5-fluorouracil, epirubicin, and cyclophosphamide) [71]. The study aimed to predict the physiological and cognitive effects of Andrographolide, *Andrographis* herb, *Eleutherococcus* root, and their fixed combination (AE) and the combination of *Rhodiola* roots, *Schisandra* berries, and *Eleutherococcus* roots (RSE). A transcriptome-wide mRNA microarray analysis of human T98G neuroglia cells revealed that FEC altered 67 genes related to neuronal development, 37 genes related to sensory system development, 12 genes related to axon extension, and 3 genes related to neuron migration. Co-incubation with *Andrographis paniculata* (AP) reduced FEC-induced dysregulation of genes associated to neuronal death and inhibition of neurogenesis, as well as 16 genes that inhibit nervous system processes. Co-incubation with AE inhibited FEC-induced deregulation of genes linked to axon extension, migration, nerve conduction, and other nervous system functions. The results indicated that adaptogenic plant extracts had beneficial effects on neuronal activities that are associated with moderate cognitive defects in cancer chemotherapy. The results suggested that The transcriptome-wide mRNA microarray profiles of neuroglial cells were significantly altered as a consequence of the combination of adaptogenic plant extracts and cytostatic medicines which further indicates beneficial impact on cognitive deficiencies induced by cancer chemotherapy [71].

## Tamoxifen

Tamoxifen is one of the most commonly used treatments for estrogen receptor positive breast cancer in younger women as it can be used regardless of age and menopausal status. Tamoxifen is an effective anti-estrogen drug that inhibits breast tumor growth, but it can also act on other estrogen receptor expressed sites including brain as it readily crosses the blood-brain barrier [108, 109]. Clinical studies have reported that tamoxifen treated patients exhibit altered cognitive ability such as immediate and delayed visual memory, verbal memory, visuo-spatial ability, significantly lower word fluency [109]. In a recent study, Galvano et al., used a mouse model of chronic tamoxifen exposure to examine the effects on the brain function. They observed a significantly increased freezing response in fear conditioning paradigm, without changes in spatial learning, locomotive activity and anxiety like behaviors. Hippocampal transcriptomic profiling revealed 669 genes significantly altered between control and tamoxifen treated groups. 647 genes were down-regulated, and 22 were up-regulated. Down regulated pathways were associated with cilium movement, microtubule assembly, regulation and transport motors (genes identified: *DNAI3*,* ODAD4*,* CFAP43*,* CFAP 206*,* ODAD2*,* FOXJ1*,* HYDIN*) while the upregulated pathways involved in negative regulation of dendritic spine morphogenesis (genes identified: *PREN*,* UBE3A*,* NGEF*,* EFNA1*,* NLGN3*,* DNM3*,* DTNBP1*,* NLGN1*). These findings suggest that tamoxifen exposure alters the genes critical for neuronal connectivity, potentially contributing to cognitive side effects observed in breast cancer treatment [76].

## Other cancer therapy regimens

Oxidative stress and pro-inflammatory pathways are one of the basic mechanisms for CICI [110]. Oppegaard et al., 2021, conducted an evaluation of differentially expressed genes and disrupted inflammatory pathways in two separate groups of cancer patients, one group reporting CICI and the other not reporting CICI [77]. The Attentional Function Index (AFI) was employed as a self-reported measure to evaluate CICI. AFI scores below 5 suggest low levels of cognitive function, whereas scores above 7.5 indicate high levels of cognitive function. Out of the 185 patients in Sample 1, 49.2% had an AFI score below 5, whereas 50.8% had an AFI value above 7.5. Out of the 158 patients in Sample 2, 50.6% had an AFI score below 5, whereas 49.4% had an AFI value above 7.5. The patients for this study were at least eighteen years old, diagnosed with lung, gastrointestinal, breast, or gynecological cancer, having undergone chemotherapy within the last four weeks, having at least two more chemotherapy cycles planned. RNA-seq was employed to analyze data from 182 patients in Sample 1. The data from 158 patients in Sample 2 was analyzed using microarray. Five of the twelve KEGG (Kyoto Encyclopedia of Genes and Genomes) signaling pathways that were substantially perturbed between the AFI groups were associated with inflammatory mechanisms, including cytokine-cytokine receptor interaction, mTOR signaling pathway, MAPK signaling pathway, IL-17 signaling pathway and tumor necrosis factor signaling [77].

Allogeneic haematopoietic stem cell transplantation (aHSCT) is the only effective cure for high-risk hematological malignancies. Post-treatment, even in individuals who have been cured, fatigue and cognitive dysfunction are common symptoms that significantly impact quality of life for many years. Various factors can contribute to central nervous system (CNS) dysfunction following allogeneic hematopoietic stem cell transplantation (aHSCT), such as the administration of neurotoxic chemotherapy and the occurrence of infections. Boberg et al., 2023, examined the immune surveillance and metabolic activities in the central nervous system (CNS) in relation to fatigue and cognitive dysfunction. Boberg et al., had employed 26 patients who had undergone allogeneic hematopoietic stem cell transplantation (aHSCT) and had been in remission for a long period of time (1–6 years post aHSCT). The study was categorized into different groups: fatigued (*n* = 13), non-fatigued (*n* = 13) and cognitive dysfunction (*n* = 7), no-cognitive dysfunction (*n* = 19). The cognitive dysfunction was exclusively observed in the fatigued group. According to the findings patients with cognitive impairment exhibited higher level of activated T-cells and NK-cells in their cerebrospinal fluid (CSF) [78]. Sequencing of CSF extracellular mRNA in all patients with fatigue showed that 76 genes were expressed differently compared to patients who did not have fatigue. The GO-term analysis indicated a decrease in apoptosis and autophagy, as well as a reduction in the production of cytokines related to immune responses and the migration of immune cells. Demonstrating cognition, 24 genes clearly altered between patients with and without cognitive impairment. From the downregulated genes in cognitive dysfunction, GO terms comprised processes essential for cell-cell adhesion, crucial to axon guidance and synaptic plasticity, as well as responses to oxidative stress, noradrenergic neuron development and negative regulation of cell death. By contrast, GO terms from the upregulated genes included in reactive gliosis, neuroinflammation response, and the extrinsic apoptotic signaling pathway in the cognitive dysfunction and fatigue group [78].

## The cancer-chemotherapy-brain interplay

As part of the Cancer Neuroscience field, studies on chemobrain require a broad perspective that considers the complex interactions between the tumor, treatment, and the nervous system. For example, brain tumors can inevitably affect brain functions [111, 112, 113] and the degree of impact may depend on the type, locations, and progression of the tumors [114, 115, 116, 117]. This baseline vulnerability makes brain cancer distinct from systemic cancers, where central nervous system is not directly compromised prior to treatment. In glioblastoma, surgery, radiotherapy, and chemotherapy may initially relieve mass effect and edema, thereby stabilizing cognition, but these interventions also carry risks of delayed neurotoxicity, including hippocampal damage and executive dysfunction. Moreover, cognitive performance independently predicts survival in glioblastoma patients, underscoring its importance for prognosis and treatment planning [118].

Brain function can also be affected by cancers outside the CNS. Apart from the mental psychological factors associated with cancer, the non-CNS cancer cells can cause systemic inflammation [119, 120] or dysregulate the gut-brain axis [121, 122]. In a resting-state fMRI study of 31 breast cancer patients, 55% exhibited cognitive impairment prior to treatment. Among these patients, 59% had persistent impairment and 41% developed late-onset impairment, suggesting that even non-CNS cancers can contribute to cognitive dysfunction independent of therapy [123]. Understanding the combined impact of each cancer type and its relevant chemotherapy is essential, as it could lead to novel neurotoxic outcomes not seen with either factor alone. Supporting this idea, a recent preclinical study demonstrated cognitive deficits only detected when cancer-bearing mice were treated with their targeted CAR-T therapy [124]. Together, these findings emphasize that future preclinical chemobrain studies should incorporate tumor-bearing models to disentangle tumor-driven versus therapy-driven effects. In the case of brain cancer, this “double-hit” paradigm where tumor-related neural compromise is further aggravated by chemotherapy represents a particularly vulnerable context that warrants dedicated mechanistic and translational research.

These vulnerabilities are further illuminated by insights from cancer neuroscience, a growing field that focuses on the dynamic, bidirectional interactions between tumor cells and neural constituents of the CNS. Rather than studying neurons, glia, or tumors in isolation, this perspective highlights the continuous communication between these components and their shared role in shaping cognitive outcomes and therapy-related side effects.

## Cancer neuroscience perspective

Tumor–CNS crosstalk is increasingly recognized as a driver of both tumor progression and neurological dysfunction. For example, glioblastoma cells release excitatory neurotransmitters such as glutamate, which disrupt neuronal signaling and contribute to seizures, cognitive decline, and network disintegration [125, 126, 127]. Tumor-derived molecules, including microRNAs like miR-26, can induce aberrant cell-cycle re-entry in neurons, leading to neurodegeneration and loss of post-mitotic stability [128, 129]. Conversely, neurons and glia can support tumor growth through synaptic activity and paracrine signaling, creating a feedback loop that integrates cancer into functional brain circuits. Microglial activation and infiltration further promote cortical hyperexcitability and tumor progression [130], while radiotherapy and chemotherapy amplify these processes by driving neuroinflammation and connectivity loss [131, 132, 133].

This bidirectional communication between tumor and CNS components highlights why brain cancer patients represent a uniquely susceptible population in the context of chemobrain. Here, treatment-related side effects cannot be viewed in isolation but must be understood within the broader framework of tumor–neuron–glia–immune interactions. Targeting these pathways whether through immunomodulation, disruption of neuron–tumor synaptic signaling, or neuroprotective interventions offers a promising avenue for mitigating cognitive decline and improving outcomes in cancer survivors.

## Future directions to study chemobrain through transcriptomics

To enhance our understanding of CICI, future research should prioritize the development of a comprehensive single-cell atlas across various brain regions subjected to different chemotherapy agents. Recent studies, such as those by Yao et al. (2023) and Zhang et al. (2023) [134, 135], have demonstrated the power of high-resolution transcriptomic and spatial cell-type atlases to reveal the complexity of cell-type diversity and spatial organization in the mouse brain. Yao et al. reported a detailed cell-type atlas created from around 7 million cells, identifying 5,322 clusters that reflect unique features of cell-type organization across different brain regions. Similarly, Zhang et al. integrated spatially resolved single-cell transcriptomics with high molecular resolution, generating over 5,000 transcriptionally distinct cell clusters.

**Fig. 2:**
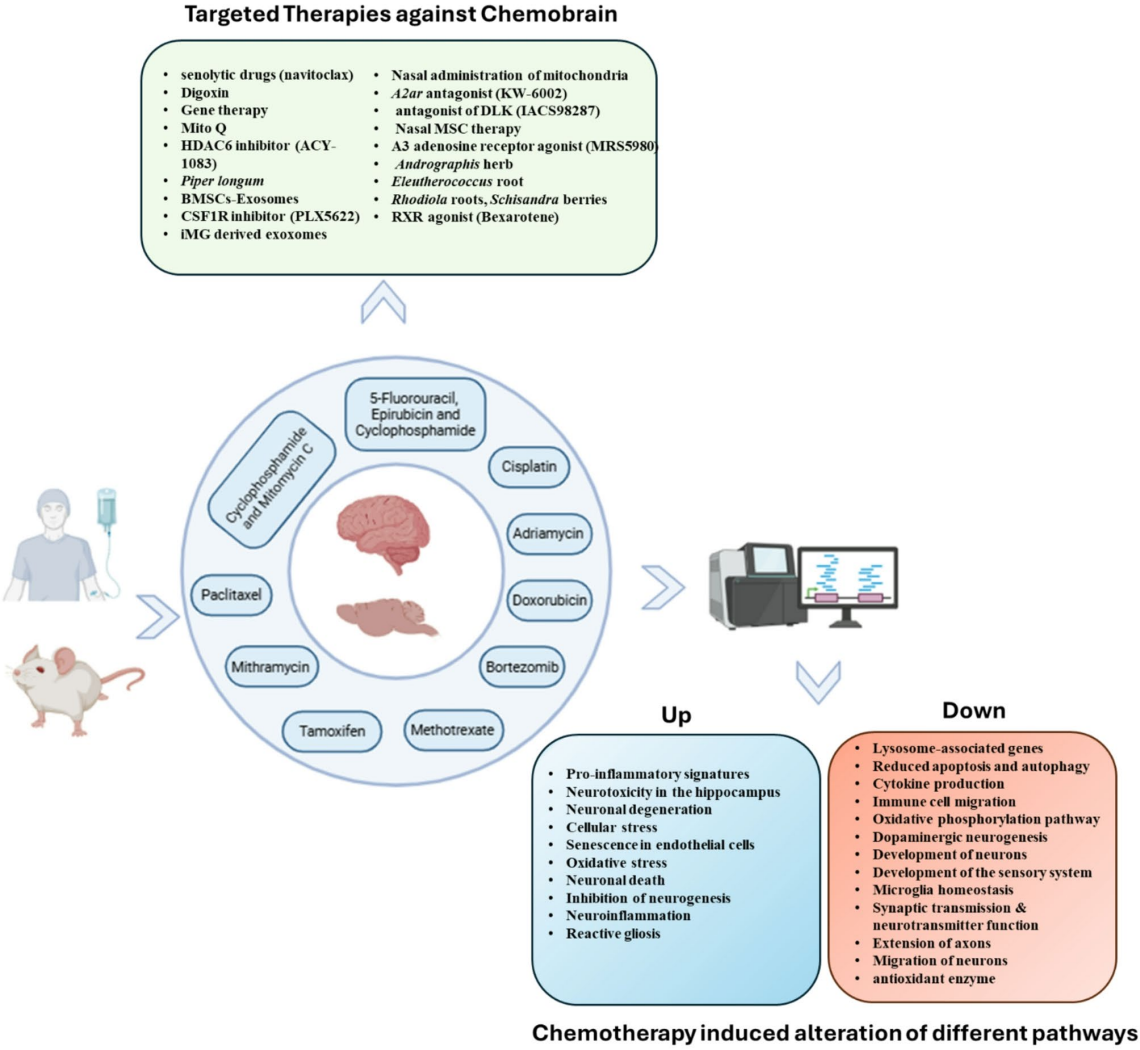
Schematic representation of the workflow depicting pathways altered by chemotherapy administration and the reported therapeutic interventions targeting these effects. (This figure has been prepared by the help of Bio render)

By leveraging these advanced methodologies, researchers can better dissect the cellular heterogeneity and identify specific cell types most affected by chemotherapeutics, providing valuable insights into the molecular pathways underlying CICI. Moreover, transcriptomic data across several chemotherapy agents, including paclitaxel, bortezomib, mithramycin, methotrexate, doxorubicin, and cisplatin, reveal overlapping mechanisms that drive CICI. These agents commonly disrupt neuroinflammatory pathways (e.g., IL-6, TNF-α, IL-23 A, and microglial activation), synaptic transmission (e.g., GABA receptors, serotonin signaling), oxidative stress (e.g., Nrf2 and mitochondrial dysfunction), autophagy, and cell cycle regulation (Fig. 2). The consistent involvement of these pathways in multiple chemotherapies suggests that they play a central role in CICI pathogenesis. Integrating spatial transcriptomics will be crucial for examining how these molecular alterations occur within specific brain regions and microenvironments, thus offering deeper insights into their contributions to cognitive dysfunction.

Studies by Langlieb et al. (2023) and Allen et al. (2023) [136, 137] have shown the potential of combining single-nucleus RNA sequencing with spatial transcriptomics methods like Slide-seq to elucidate the intricate interactions between cell types within neuroanatomical structures. By mapping gene expression profiles in situ, researchers can gain insights into how chemotherapy affects neuronal and glial interactions, contributing to the overall pathology of chemobrain. This will aid in identifying biomarkers and therapeutic targets, ultimately leading to more effective strategies for mitigating the cognitive deficits associated with cancer treatment. Future research should focus on targeting these common pathways such as neuroinflammation, mitochondrial dysfunction, and autophagy as potential therapeutic strategies to alleviate CICI and improve the quality of life for cancer survivors (Fig. 2).

## Expanding toward spatial multi-omics integration

Building on these advances, the integration of spatial transcriptomics with spatial proteomics offers an even more comprehensive view of chemobrain mechanisms. While spatial transcriptomics preserves the spatial context of gene expression [138, 139], spatial proteomics complements this information by capturing protein abundance, post-translational modifications, and signaling pathway activation within the same tissue architecture [140, 141]. Together, these spatially resolved datasets can uncover region-specific molecular signatures, reveal localized glial activation and synaptic remodeling, and identify microenvironmental niches most affected by chemotherapy. Such high-resolution, multi-omic mapping will be invaluable for defining precise therapeutic targets and developing region-specific intervention strategies.

## Conclusion

In summary, this review highlights a host of mechanisms underlying cognitive decline as a result of various chemotherapeutic strategies. It also underscores the critical need to understand the molecular mechanisms underlying chemotherapy-induced cognitive impairment through transcriptomic analyses. The evidence presented highlights the significant alterations in gene expression across various chemotherapy agents and their implications for brain function. By leveraging advanced transcriptomic techniques, including RNA sequencing, cDNA microarrays, and single-cell analyses, researchers are beginning to unravel the intricate biological pathways contributing to CICI. Despite the progress made, further investigations are required to fully elucidate the complex interplay between the various chemotherapy-induced cellular defects leading to cognitive dysfunction, including mitochondrial dysfunction, neuroinflammation, oxidative stress, and many other receptor pathways.

More attention should be focused on the prevention and treatment of cognitive decline in cancer patients by investigating the possibility of applying the preclinical data on powerful cellular and molecular therapeutics to counteract the development of cognitive decline in clinical populations, especially using those therapeutics of alternative use. Ultimately, integrating findings from transcriptomic studies with clinical insights will facilitate the development of targeted interventions aimed at preserving cognitive health in cancer survivors, ensuring a better quality of life for this vulnerable population.

## Data Availability

No datasets were generated or analysed during the current study.

## Abbreviations

KEGG: Kyoto Encyclopedia of Genes and Genomes
GO: Gene Ontology
MTX: Methotrexate
CICI: Chemotherapy-induced cognitive impairment
ADR: Adriamycin
MTR: Mithramycin
